# Cross-linking manipulation of waterborne biodegradable polyurethane for constructing mechanically adaptable tissue engineering scaffolds

**DOI:** 10.1093/rb/rbae111

**Published:** 2024-09-02

**Authors:** Nan Sheng, Weiwei Lin, Jingjing Lin, Yuan Feng, Yanchao Wang, Xueling He, Yuanyuan He, Ruichao Liang, Zhen Li, Jiehua Li, Feng Luo, Hong Tan

**Affiliations:** College of Polymer Science and Engineering, State Key Laboratory of Polymer Materials Engineering, Med-X Center of Materials, Sichuan University, Chengdu 610065, China; College of Polymer Science and Engineering, State Key Laboratory of Polymer Materials Engineering, Med-X Center of Materials, Sichuan University, Chengdu 610065, China; College of Polymer Science and Engineering, State Key Laboratory of Polymer Materials Engineering, Med-X Center of Materials, Sichuan University, Chengdu 610065, China; College of Polymer Science and Engineering, State Key Laboratory of Polymer Materials Engineering, Med-X Center of Materials, Sichuan University, Chengdu 610065, China; Department of Neurosurgery, West China Hospital, Sichuan University, Chengdu, Sichuan 610000, China; Laboratory Animal Center, Sichuan University, Chengdu 610041, China; College of Polymer Science and Engineering, State Key Laboratory of Polymer Materials Engineering, Med-X Center of Materials, Sichuan University, Chengdu 610065, China; Department of Neurosurgery, West China Hospital, Sichuan University, Chengdu, Sichuan 610000, China; College of Polymer Science and Engineering, State Key Laboratory of Polymer Materials Engineering, Med-X Center of Materials, Sichuan University, Chengdu 610065, China; College of Polymer Science and Engineering, State Key Laboratory of Polymer Materials Engineering, Med-X Center of Materials, Sichuan University, Chengdu 610065, China; College of Polymer Science and Engineering, State Key Laboratory of Polymer Materials Engineering, Med-X Center of Materials, Sichuan University, Chengdu 610065, China; College of Polymer Science and Engineering, State Key Laboratory of Polymer Materials Engineering, Med-X Center of Materials, Sichuan University, Chengdu 610065, China

**Keywords:** waterborne polyurethane, modulus, mechanical adaptation, tissue engineering scaffold, central nervous repair

## Abstract

Mechanical adaptation of tissue engineering scaffolds is critically important since natural tissue regeneration is highly regulated by mechanical signals. Herein, we report a facile and convenient strategy to tune the modulus of waterborne biodegradable polyurethanes (WBPU) via cross-linking manipulation of phase separation and water infiltration for constructing mechanically adaptable tissue engineering scaffolds. Amorphous aliphatic polycarbonate and trifunctional trimethylolpropane were introduced to polycaprolactone-based WBPUs to interrupt interchain hydrogen bonds in the polymer segments and suppress microphase separation, inhibiting the crystallization process and enhancing covalent cross-linking. Intriguingly, as the crosslinking density of WBPU increases and the extent of microphase separation decreases, the material exhibits a surprisingly soft modulus and enhanced water infiltration. Based on this strategy, we constructed WBPU scaffolds with a tunable modulus to adapt various cells for tissue regeneration and regulate the immune response. As a representative application of brain tissue regeneration model in vivo, it was demonstrated that the mechanically adaptable WBPU scaffolds can guide the migration and differentiation of endogenous neural progenitor cells into mature neurons and neuronal neurites and regulate immunostimulation with low inflammation. Therefore, the proposed strategy of tuning the modulus of WBPU can inspire the development of novel mechanically adaptable biomaterials, which has very broad application value.

## Graphical Abstract

**Figure:**
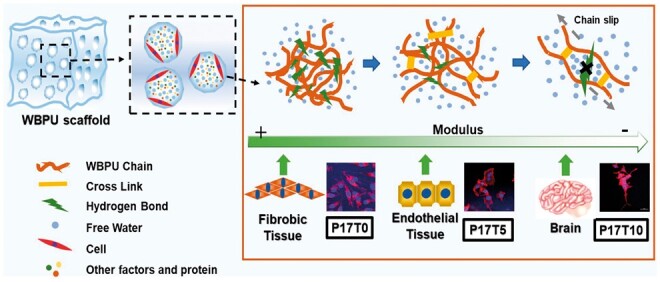

## Introduction

The design principle of tissue engineering scaffolds assumes mimicking the extracellular matrix (ECM) of native tissue to the highest possible extent. Cells receive different signals from surrounding microenvironments, such as biochemical, mechanical and topographical cues [1, 2]. Increasing evidence has demonstrated that mechanical cues are critically important in regulating cellular functions and even tissue regeneration [3–8]. Cells are very sensitive to the mechanical properties of surrounding environments and can convert mechanical stimuli into biochemical signals, exhibiting a wide range of effects on cell behavior [8–14]. Tissue engineering scaffolds with appropriate mechanical properties can generate corresponding forces on endogenous cells to mediate cell deformation and secretion of ECM, trigger cell self-renewal ability and even decisively direct cell differentiation [3]. For example, central nervous tissue is the softest tissue in the body, and it is extremely hard to repair because of its sophisticated functions and limited regeneration [15]. Studies have demonstrated that the stiffness of scaffolds can affect the adhesion, migration and proliferation of neural stem cells, stimulate the regeneration of damaged axons and induce the differentiation direction toward neurons or glial cells [16–18]. Ali *et al.* [17] found that the neural progenitor cells (NPCs) of rats exhibit better adhesion on soft (2 kPa) than on hard (35 kPa) gelatin-based hydrogels. However, when the stiffness of the scaffold was below 100 Pa, the dispersion and adhesion of neural stem cells were inhibited, indicating that overly soft materials may also negatively affect the repair of central nerves. Therefore, mechanically adaptable scaffolds that match cell adhesion and proliferation are critical to central nervous tissue repair.

The stiffness of implanting materials also affects the inflammatory response of a host. Inflammatory response to a certain degree is indispensable for the biomaterial’s tissue integration with the scaffold, but an aggressive or chronic inflammatory response leads to implant failure [19, 20]. Recent reports have demonstrated that tuning the matrix stiffness can regulate macrophage behavior and further inflammatory response. Chuang *et al.* [21] reported that softer substrates drove J774A.1 macrophages toward the proinflammatory M1-like phenotype and triggered more cytoplasmic reactive oxygen species in regulating inflammation compared to stiffer substrates. Li *et al.* [22] also demonstrated that stiffer substrates enhanced the anti-inflammatory production of nitrites from native and classically activated RAW 264.7 macrophages. In contrast, Zhuang *et al.* [23] reported that stiffer surfaces induced a more severe inflammatory response due to more macrophages polarized into the M1 phenotype. These contradictory results may originate from the difference between tissue-derived macrophages and the components of substrates. In traumatic brain injury (TBI), neuroinflammation is primarily mediated by activated microglia. Beyond the limited regeneration capacity of adult brains, the activation and duration of destructive inflammation after trauma are the primary reasons for the failure of brain nervous regeneration [19, 24]. Thus, scaffolds require a favorable mechanical cue, such as an adaptable modulus, to regulate the inflammatory response and avoid destructive inflammation.

The matrix stiffness of polymers usually depends on the cross-link density, polymer concentration and the presence of a condensed structure (e.g. crystallization) [25, 26]. Polymers with high cross-link density, concentration and crystallinity exhibit an increased stiffness. For example, increasing the content of cross-linking agents usually leads to an increased modulus of polymer substrates [27, 28]. From another perspective, water is an effective regulator of mechanical properties for polymers, and the interchain hydrogen bonds of polymers might be destroyed by invasive water molecules. Especially the hydrated network of polymer hydrogel resembles the ECM of soft tissues, and the low modulus of the central nervous system can be easily approached through simple composition regulation [29]. Therefore, hydrogel materials are broadly used for soft tissue repair [30–34]. However, their application is limited by poor structural and mechanical stability, as well as very small nanoscale pores with insufficient size, which are inadequate for cell permeability during the process of long-term repair process *in vivo*. Mechanically and structurally stable hydrogels are usually obtained by increasing their cross-link density or polymer concentration, accompanying with an increased stiffness and decreased pore size [25]. Thus, alternative scaffolds for soft tissue repair are still under exploration. Solid 3D scaffolds are controllable and can be produced with high reproducibility, offering long-term structural and mechanical stability, and their size and porosity can be flexibly adjusted [35, 36]. Large microscale pores of porous scaffolds allow deeper and more uniform nutrient transport, enabling free cellular movement throughout the internal structure without considerable resistance [37]. However, most solid 3D scaffolds are stiff and have low hydrophilicity to maintain the structural stability so that it is very difficult to satisfy the biophysical requirements preferred by soft cells. Therefore, soft polymer matrixes with water infiltration ability are highly desired.

Polyurethane typically consists of soft and hard segments, with the thermodynamic incompatibility between these segments resulting in microscopic phase separation within the material. The hard segments with higher glass transition temperatures typically exhibit strong intermolecular forces, serving as physical crosslinks that impart substantial modulus and strength to the material. The inclusion of soft segments further endows polyurethane with outstanding elasticity. Consequently, the mechanical properties of polyurethane are closely linked to the extent of its microphase separation. Waterborne polyurethanes, a special polyurethane with favorable hydrophilicity and biocompatibility, can meet the biophysical requirements for preparing soft scaffolds [38–41]. In our previous reports [42, 43], a series of waterborne biodegradable polyurethane (WBPU) scaffolds were fabricated for tissue engineering regeneration. For example, the 3D WBPU scaffolds prepared by emulsion freeze-drying showed favorable nerve regeneration performance with axonal regeneration, synaptic reconstruction and functional motor recovery in TBI. However, there are remaining issues to be solved in the repair process, particularly the mismatch of modulus and the biodegradation rate of the engineered scaffolds compared to the injured tissue to induce severe immunostimulation are crucial. Since the mechanical properties of WBPU strongly depend on the phase separation of the polymer segments and interchain hydrogen bonds, a facile and effective strategy is required to manipulate them [44].

In this work, it was found that the modulus of WBPU scaffolds can be adjusted by the cross-linking manipulation of phase separation and water infiltration. Herein, aliphatic polycarbonate and trimethylolpropane (TMP) were introduced to a polycaprolactone (PCL)-based WBPU to interrupt the crystallization and enhance covalent cross-linking. The modulus of WBPU scaffolds exhibited an abnormal gradual decrease with the cross-linking extent. Different cells could adapt to the gradient modulus of WBPU scaffolds for various tissue regeneration. Especially, the scaffolds with a relatively higher cross-linking extent and less microphase separation could absorb more water to form relatively soft hydrated scaffolds for matching the softness of central nervous tissue repair. We further demonstrated that the superior application of the scaffolds for the in vivo brain tissue regeneration model compared with traditional biodegradable PU hydrogels.

## Experimental section

### Materials

Lysine diisocyanate (LDI, analytical pure); 1,3-propanediol (PDO, analytical pure) was distilled under reduced pressure before used; polyethylene glycol (PEG, analytical pure, Mn = 1450 g/mol) was bought from Dow Chemical Company; PCL (analytical pure, Mn = 2000 g/mol), was bought from Daicel, Japan; polycarbonate (PCDL, analytical pure, Mn = 1000 g/mol) was brought from TOSOH Corporation; L-lysine (chain extender, analytical pure), Si Chuan Emeishan Ronggao Biochemical Products Co. Ltd, China; TMP (analytical pure); PBS (analytical pure, Shanghai Kexing Trading Co., Ltd.), CCK8 kit and ELISA kit (Biyun Tian); MEM-basic (Hyclone) calf serum (Gibco); penicillin, tomycin, paraformaldehyde, lipopolysaccharide (LPS), 4’,6-diamidino-2-phenylindole (DAPI) and other fluorescent dyes (medical grade, Solarbio); anhydric ethanol, acetone and other common chemical reagents (analytical pure, Chengdu Kelon Chemical Reagent Co., Ltd.); pancreatic enzyme (Gibco); All cells used in the experiment were donated by West China Hospital of Sichuan University.

### Preparation of WBPU emulsion

The WBPU emulsion was synthesized modified from our previous report [45]. Herein, a series of WBPUs with various contents of cross-linker TMP was synthesized (Table 1), and they are named PxTy, where x represents the PEG molar ratio in soft segments, and y represents the wt.‰ content of TMP. The synthesis process of WBPU is shown in Supplementary Figure S1. Firstly, PCL, PEG and PCDL were dehydrated in a three-neck flask with mechanical stir at 90°C for 1.5 h. Under the protection of N_2_, LDI and 0.1wt% catalyst organic bismuth were added to the flask and reacted at 80°C for 1.5 h. Then, PDO and TMP were added and the chain was extended at 70°C for 1 h. Secondly, the prepolymer was dissolved by appropriate acetone to reduce the viscosity and slowly add concentrated lysine solution for reacting 5 min. Thirdly, the solution was emulsified for 1.5 h at a high speed of 1500 r/min, then the rotating speed was reduced to 500 r/min and the equilibrium emulsion was stabilized for 30 min to obtain the waterborne polyurethane emulsion.

**Table 1.** The components of WBPUs

Sample (PxTy)^a^	Molar ratios of LDI/soft segment/chain extender	Molar ratio of NCO/(–OH and –NH_2_)	Molar ratio of PEG/soft segment (%)	Molar ratio of PCDL/soft segments (%)	TMP ratio (wt%)	Molar ratio of (PDO+TMP)/lysine
P25T0	3.6:1:2	1.2:1	25	0	0	1:1
P17T0	3.6:1:2	1.2:1	17	10	0	1:1
P17T5	3.6:1:2	1.2:1	17	10	0.5	1:1
P17T10	3.6:1:2	1.2:1	17	10	1	1:1
P17T15	3.6:1:2	1.2:1	17	10	1.5	1:1

a PxTy: the ‘x’ represents the PEG molar ratio in soft segments and the ‘y’ represents the wt.‰ content of TMP.

### Preparation of the PxTy scaffolds

WBPU emulsion with solid content of 14% was prepared and injected into the mold with pipette. The mold was placed in a 4°C environment for 4 h, then placed in a −24°C environment for 24 h. The scaffolds were prepared by a freeze-drying machine for 24 h, and finally moved to an electric thermostatic drying oven for 24 h before being taken out and stored at room temperature. All of the scaffolds were sterilized with gamma irradiation using doses of 25 kGy before experimentation and testing.

### Preparation of PxTy film

The synthesized PxTy emulsion was poured into a glass surface dish, and the water and solvent were dried at 45°C in a drying oven until a dry film was formed. The wet polyurethane film was obtained by soaking the dry film in PBS and placing it in a shaker at 37°C and 90 r/min for 24 h.

### Preparation of PU hydrogel

To obtain PU hydrogel, unsaturated double bond was imported and the amount of hydrophilic PEG was increased, compared with WBPU scaffolds. In brief, 5 mmol PCL (550 g/mol) and 15 mmol PEG (Mn = 1450 g/mol) were added in 150-ml three-neck flask to decompression dehydration at 95–100°C for 2 h. After reducing the temperature, 31.2 mmol L-lysine diisocyanate (LDI, purified by vacuum distillation before use) and catalyst (0.1 wt% organo-bismuth) were added and reacted at 78–80°C for 1 h. Reducing the reaction temperature by condensation reflux, appropriate amount of acetone was added to reduce the viscosity of the system. Then, 6 mmol 2,2-dihydroxymethylbutyric acid was added to chain extend for 2 h. Subsequently, 12 mmol hydroxyethyl methacrylate was added to react with the remaining isocyanate for 2 h. A 10 wt% sodium hydroxide solution was added dropwise through a syringe to neutralize the pH. After cooling to room temperature, a certain amount of deionized water was added to reverse phase emulsification. The aqueous PU solution was prepared by removing acetone from the system and the solid content of PU solution was adjusted to 20 wt%. After removing bubble, the PU solution with adding 0.1 wt% photoinitiator I2959 was reacted by UV light (365 nm) for 5 h to obtain the PU hydrogel. The modulus of the PU hydrogel is 7.5 ± 1.2 kPa.

### Equilibrium water content of PxTy film

The mass of the dried polyurethane film was recorded as *m_0_*, immersed the film in PBS and placed it in a shaker with a shaking rate of 90 r/min at 37°C. When the set time was reached, take the film out and wipe the water on its surface with filter paper, then record the mass of the moisture film as *m_1_*. Equilibrium water content (*W*) was calculated by the following formula (1) (*n* ≥ 3).

(1) $$W=\frac{m_{1}-m_{0}}{m_{1}}\times 100\%$$

### Surface infrared spectroscopy (ATR-FTIR)

The dry and wet WBPU films prepared according to the above methods were directly characterized by ATR-FTIR, and the data were collected by Nicolet560 infrared spectrometer with a collection range of 4000 ∼ 600 cm^−1^. The resolution was 4 cm^−1^ with 32 scans.

### Scanning electron microscopy

The previously prepared scaffolds were cryofractured in liquid nitrogen. The fracture surface of the scaffold was sprayed with gold and observed by the Inspection F50 Electron Microscope (FEI Company, USA). The pore size, pore distributions and porosity were calculated by Image J.

### Swelling degree of the scaffolds

The mass of the dried scaffold was recorded as *m*, immersed the scaffold in PBS and placed it in a shaker with a shaking rate of 90 r/min at 37°C. When the set time is reached, take the scaffold out and wipe the water on its surface with filter paper, then record the mass of the swelled scaffold as *m′*. Swelling degree (*Q*) of the scaffolds was calculated by the following formula (2) (*n* ≥ 3).

(2) $$Q=\frac{m^{\prime }-m}{m}\times 100\%$$

### Mechanical properties of the scaffolds

The mechanical properties of the scaffolds were measured by the HZ-1004 universal tension machine (Dongguan Lixian Instrument Scientific Co. Ltd). The test sample size was 1.0 × 1.0 × 0.3 cm^3^. The test temperature was 25°C and the compression speed was 10 mm/min (*n* ≥ 3).

### Differential scanning calorimetry

TA DSC250 instrument was used for thermal analysis of the scaffolds. The samples both saturated with water and extracted water from the pores were tested. During the test, the temperature was firstly cooled to −50°C and hold for 3 min, then heated to 180°C at 10°C/min. All tests were performed under a nitrogen atmosphere by using the samples with masses between 5 and 10 mg.

### Cell viabilities in co-culturing process with samples

BV2 cells were purchased from HuaAn Biological Company, PC12 cells were purchased from SaiLi biological company.

First, the scaffolds were sterilized with gamma radiation using doses of 25 kGy. Then, they were soaked overnight in an incubator with PBS. Before the cells were inoculated, the samples were placed into a 48-well plate. In subsequent, the samples were rinsed with PBS and complete culture medium both of two times. All cells were cultured in a 5% CO_2_ and 37°C incubator. About 50 000 cells/well and 1 ml medium/well were added to a 48-well plate with the sample. Among them, the culture medium of PC12 cells contained 50 ng/ml NGF. BV2 cells adhered after 24 h, and the cell culture medium was replaced with a complete culture medium containing LPS or LPS-free. After the predetermined culture time, the culture medium was sucked out, the sample was washed twice with sterilized PBS and the residual culture medium was removed. The cell culture time was 1 day and 6 days, respectively. Similar method is also used to co-culture the cells with the WBPU films.

### Effects of the complete degradation solution on PxTy on BV2 cells

The BV2 cells in the culture dish were digested by trypsin, centrifuged and mixed into the medium to form cell suspension. The cell concentration in the suspension was estimated by red blood cell counting board. Cells were seeded to the bottom of the 96-well plate, 5 × 10^3^ for each well. After cell adhesion for 24 h at 37°C, the medium was removed and 0.15 ml of the complete degradation solution of PxTy with different concentrations was transferred into each well. After cells adhered for 24 h, the cell culture medium was replaced by a complete culture medium containing LPS or LPS-free. After culturing for 24 h, the relative cell number was detected by MTT method and the cell survival rate was obtained by comparing the measured O.D. value of degradation fluid cells with that of control cells. Three parallel samples were set for each concentration, and the results obtained were the average values of the three parallel samples.

### Confocal laser-scanning microscope

After the specified culture time, the samples were removed and fixed with paraformaldehyde for 45 min, then were washed three times with sterile PBS to remove the adsorbed paraformaldehyde in the material. After staining with DAPI (blue) and phalloidin (red), the number and morphology of cells on the samples were observed under a confocal laser-scanning microscope (Nikon N-SIM).

### Western blot

After PC12 cells were cultured on the samples (PxTy films) for 6 days, the sample was removed with tweezers and the cells on the sample were collected by cell scraping. After centrifugation, the supernatant was removed. Cell lysis was performed to balance the protein concentration and obtain the protein specimens of each sample.

After preparing the separation glue, it was added to the two glass plates and sealed with water for 45 min. Then, the water was poured off, a comb was inserted and wait for gelation. Carefully pull out the comb and wash the injection hole with electrophoresis buffer. Fix the glass panel to the buffer room and add appropriate electrophoresis buffer. Sample with equal volume was added to each sample hole and a small amount of marker was added on the edge. The electrophoresis buffer covered up the glass plate. During electrophoresis, it was stopped when marker appeared at the lowest molecular weight band. In sequence, use sandwich wrap and ice bath. Slightly rinsed the PVDF membrane and then added the sealing solution to seal for 2–3 h. After cleaning, add the primary antibody and put in 4°C environment overnight. The next day, remove and add the secondary antibody, color rendering.

### Immunofluorescence staining

The samples were washed with PBS and fixed with paraformaldehyde for 15 min. After cleaning, Triton-100 was added and treated at room temperature for 5 min. Seal with 10% sheep serum for 30 min. Then, sheep serum was absorbed and the diluent first antibody was added, incubating in the incubator for 1.5 h. After picking out, the sample was washed with PBS several times, added the diluent secondary antibody and incubated in the incubator for 1 h. And then the secondary antibody was sucked out and stained with phalloidin and Dapi. After dyeing, it was wrapped in aluminum foil and stored at 4°C for observation.

### ELISA test

Concentrations of TNF-alpha and anti-inflammatory cytokine IL-10 released by BV2 cells on PxTy scaffolds were determined by ELISA. 2 × 10^5^ BV2 cells were inoculated on the scaffolds, and the culture plate (TCPs) was used as control. After the cells were cultured in accordance with the above methods for a specified time, the cell culture supernatant was taken and tested in accordance with the ELISA kit instructions (*n* ≥ 3).

### Animal experiments and surgical procedures

All animal protocols were approved by the Institutional Animal Care and Use Committee (IACUC) of Sichuan University (Grant 20211506A). Adult female Sprague–Dawley rats (250–300 g, Institute of Laboratory Animal of Sichuan Academy of Medical Sciences) were employed (This experiment is not gender related). Animals were anesthetized with isoflurane, followed by fixation on a stereotaxic apparatus. After disinfection and skin cutting, the superficial location of the rat motor center zone was located by using stereotaxic coordinates. A circular bone window with a diameter of 8 mm was located on the left side of the rat. Then, craniotomy was carried out by using electric surgical drills, after which dura and 5 mm × 5 mm × 4 mm brain tissues were removed through microsurgery (Zeiss, Germany). After injury, the tailored BWPU scaffolds or hydrogel were implanted into the lesion cavity. The control group was filled with sterilized PBS. Finally, the muscles, galea aponeurotica and skin were sutured in layers. After the operation, the rats were freely housed at a temperature of 23 ± 2°C and relative humidity of 50–60% with standard laboratory chow and distilled water. Animals were sacrificed 14-day post-injury for immunohistochemistry analysis. To evaluate the functional recovery of the rats after post-injury on 8 weeks, motor function scores were assessed using the Bederson scale [46]. Scores range from 0 for no observable defects, 1 for flexed forepaw, 2 for inability to resist lateral push without circling, 3 for circling.

### Tissue processing and immunohistochemistry

Animals were anesthetized before sacrifice. The samples for immunohistochemistry were perfused intracardially with PBS, followed by 4% paraformaldehyde in 0.1 M phosphate buffer at pH 7.4. Then, the scaffold and surrounding brain tissue were dissected and fixed in 4% paraformaldehyde for 24 h and cryoprotected in a graded series of sucrose solutions. Samples were cut transversely into 20-μm thick sections by a cryostat microtome (Leica, USA) and thaw-mounted onto glass slides (Fisher Scientific). For immunohistochemistry, slides were permeabilized in the permeabilizing solution 0.3% Triton X-100 (Sigma, USA) for 15 min, followed by blocking in immunostaining blocking buffer (B600060, Proteintech, USA) for 60 min at room temperature. Then, sections were incubated with a primary antibody overnight at 4°C. These primary antibodies were anti-MAP2 (Abcam, ab11267), anti-GFAP (Abcam, ab4674), anti-SYP (Abcam, ab8049), anti-DCX (Abcam, ab153668), anti-βIII tubulin (Abcam, ab18207), anti-CD86 (Invitrogen, PA5-88284), anti-CD206 (Abcam, ab64693) and anti-Iba1 (WAKO, NCNP24). After washing three times, slides were incubated with appropriate Alexa Fluor^®^ 647 and Alexa Fluor^®^ 488-conjugated secondary antibodies at 37°C for 1 h, and nuclei were stained with DAPI (0.5 g/ml) for 10 min at room temperature. Slides were cover-slipped and mounted with antifade mounting medium (Beyotime, China), and images were acquired using a confocal laser-scanning microscope (Nikon A1R MP+).

### Statistical analysis

The data are expressed as mean ± SD. Each value was an average of at least three parallel experiments. Statistical analysis between data was conducted by one-way or two-way analysis of variance (ANOVA), where *P < 0.05 was considered as a significant difference between samples, **P < 0.01, ***P < 0.001, ****P < 0.0001 were considered to be highly significant; # was considered as no significant.

## Results and discussion

### Strategy of tuning the stiffness of WBPU

**Figure 1.**
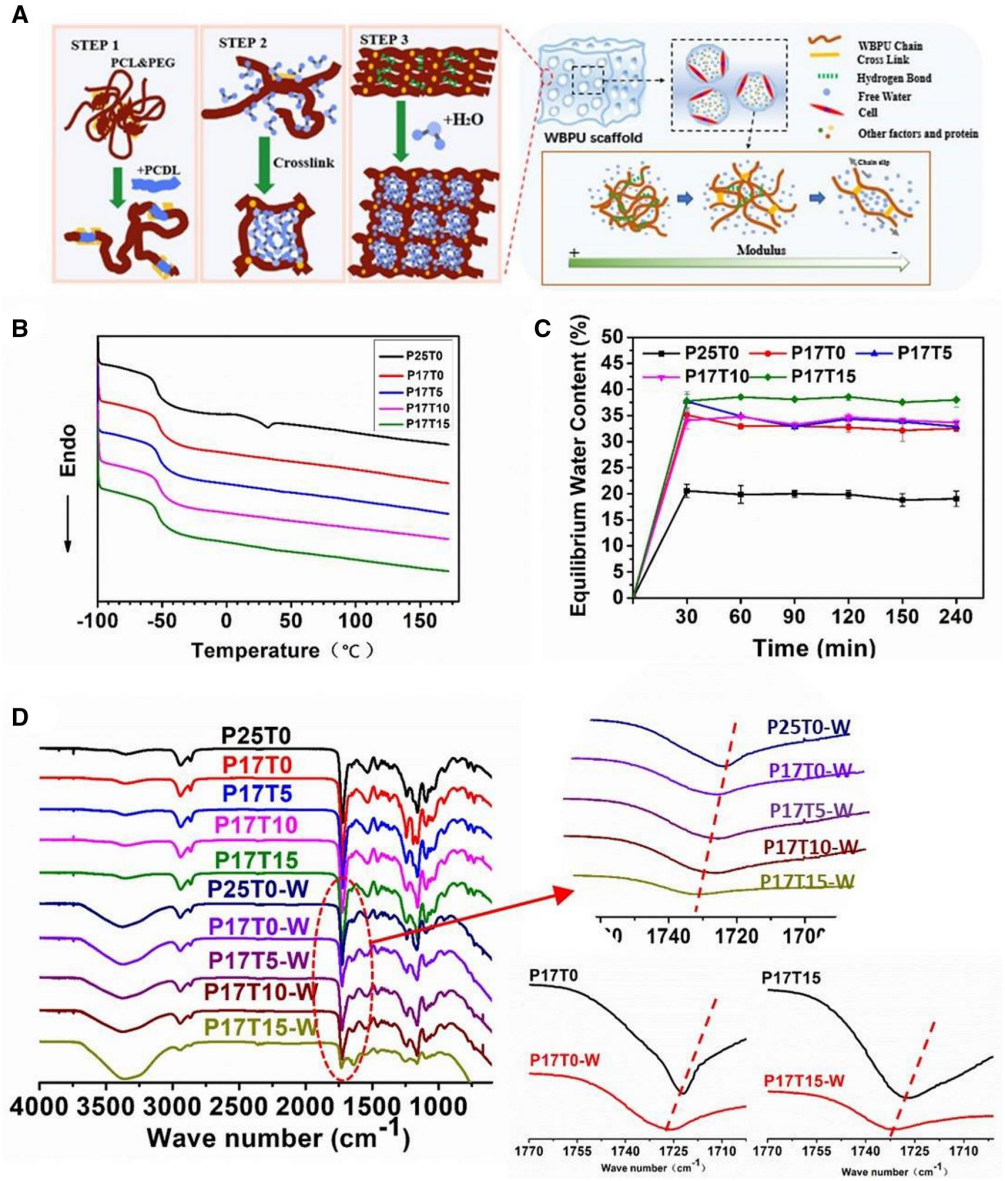
(**A**) Schematic representation of tuning the stiffness of waterborne biodegradable polyurethane (WBPU) and the mechanically adaptable mechanism of 3D porous WBPU scaffolds for regulating cell behavior. (**B**) DSC curves (in heating) of WBPU films; only the P25T0 film without PCDL and TMP exhibits a melting peak at ∼25°C. (**C**) Water absorption process of WBPU films. (**D**) ATR-FTIR spectra of the PxTy films in dry and wet states. The W in PxTy-W designates the wet state.

To manipulate interchain hydrogen bonds and phase separation for tuning the stiffness of WBPU, we performed a facile strategy to modify our previous procedure of the WBPU synthesis (Figure 1A and Supplementary Figure S1). Briefly, amorphous aliphatic polycarbonate diol (PCDL) was introduced into the polymer chain to partly replace PCL, thereby reducing the regularity of chains and inhibiting crystallization (STEP 1). Meanwhile, trifunctional TMP was introduced to increase the density of covalent cross-linking, to restrain phase separation and improve structural stability (STEP 2). After this modification, water could more easily infiltrate into the segments of WBPU to decrease the modulus while retaining a stable framework because of crosslinking (STEP 3). Notably, aliphatic polycarbonate (PCDL) was introduced into the polymer chain to interrupt the crystallization of PCL, thereby the melting peak of PCL at 25°C is absent in the series of P17Ty samples compared with the P25T0 sample that does not contain PCDL but possesses the similar PCL proportion (Figure 1B). In addition, the water absorption ability of the P17Ty samples is enhanced (Figure 1C), indicating that the interchain hydrogen bonds of the segments in P17Ty samples might weaken via the introduction of PCDL. Meanwhile, FTIR curves show that the typical vibration peaks of carbonyl are shifted to higher wavenumbers as the amount of cross-linker TMP increases, indicating a lower amount of hydrogen-bonded carbonyl groups in highly cross-linked samples (Figure 1D). Moreover, an obvious blue shift of peaks at 1720 cm^−1^ for P17Ty samples in the wet state appears, indicating that the water molecules act as plasticizers that break the hydrogen bonds in the PU segments. Thus, either adding cross-linker TMP or absorbing water can destroy the hydrogen bonds in the P17Ty samples. Based on the above strategy, mechanically adjustable WBPU scaffolds with special water-soften features are fabricated to regulate cell behavior (Figure 1A).

### Characterization of the WBPU scaffolds

**Figure 2.**
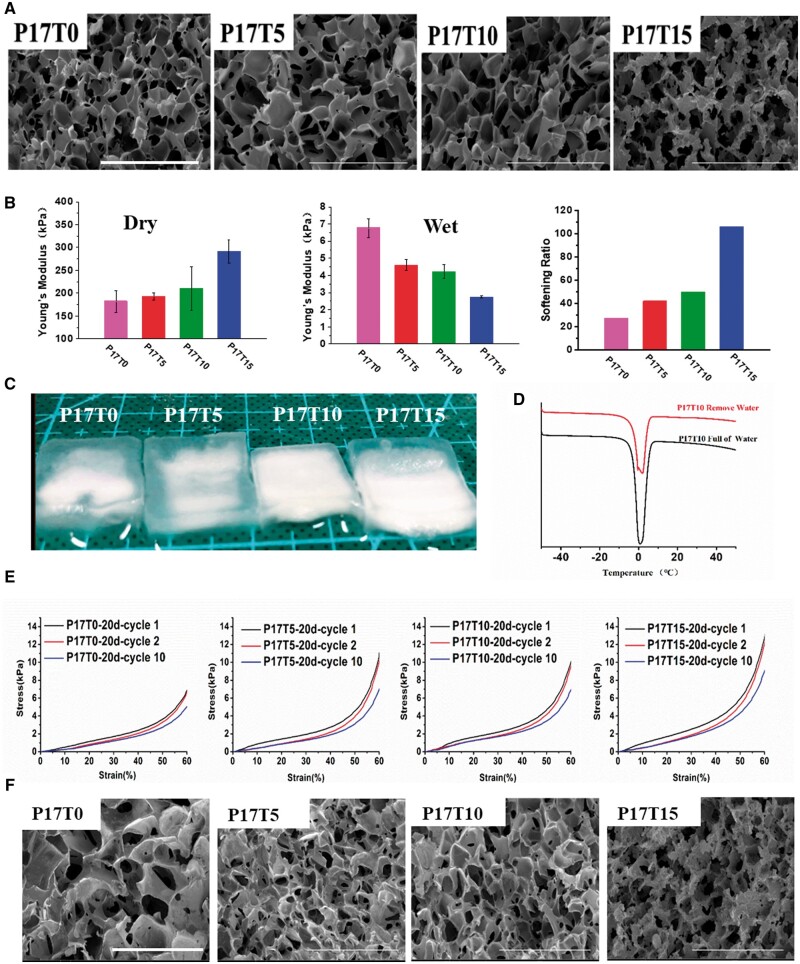
(**A**) 3D Porous microstructures of P17Ty scaffolds. (**B**) Young’s moduli of P17Ty scaffolds in dry/wet states and the corresponding softening ratios. (**C**) Photographs of P17Ty scaffolds after water absorption. (**D**) Typical heating curves of P17T10 water-saturated and water-unsaturated scaffolds. (**E**) Cyclic compressive curves of P17Ty scaffolds after immersion in PBS for 20 days. (**F**) Porous microstructures of PxTy scaffolds after immersion in PBS for 20 days and 10 times cyclic compression.

To focus on the effect of cross-linking on the properties of WBPUs, we prepared a series of 3D porous P17Ty scaffolds with high porosity (∼80%) and uniform pore size (∼95 μm) by emulsion freeze-drying, as shown in Figure 2A and Supplementary Figure S2. Such 3D porous structures of the scaffolds facilitate the transport of nutrients and metabolic products and the communication, migration and growth of cells. Meanwhile, Young’s moduli of P17Ty scaffolds in the dry state increase from 182 to 291 kPa with increasing the amount of the TMP cross-linker (Figure 2B and Supplementary Figure S3A), exhibiting strong mechanical properties with a stable framework. Intriguingly, Young’s moduli of P17Ty scaffolds dramatically decrease to less than 10 kPa for all the scaffolds after water absorption. In particular, the moduli rapidly decrease with an increase in the cross-linking degree from 6.8 kPa for P17T0 to 2.7 kPa for P17T15. Thus, the softening ratio, i.e. the ratio of Young’s modulus of sample in the dry state and that in the wet state, shows an abnormal increase with the cross-linking degree after water absorption. The maximal softening ratio for P17T15 is up to 105. In addition, the scaffolds show strong water absorption with a swelling degree of 7 0 0∼900% (Figure 2C and Supplementary Figure S3C). Remarkably, the water in scaffolds is mainly presented as free water. The absorbed water can be released from the scaffold under compression. By comparing the change of the melting peak in the differential scanning calorimetry (DSC) curve around 0°C between the water-saturated scaffold and the unsaturated scaffold (water removed by filter paper), the state of water in the scaffold can be estimated. As shown in Figure 2D and Supplementary Figure S3D, when the scaffold is water-saturated, the enthalpy of the P17Ty scaffold with a water content of about 90 wt.% is around 300 J/g, which is very close to the enthalpy of the melting of water (334 J/g [47]), and there is only one peak at 0°C. However, after the water is removed from the pores by filter paper, an extra peak appears slightly below 0°C. According to the literature [47, 48], the melting peak of free water after freezing appears at 0°C, and the melting peak of water that interacted with the molecular chain is slightly below 0°C after freezing. According to the DSC curves, most of the water stored in the scaffold is free water and can be used by cells. In the scaffold, cells mainly grow on the walls and migrate and communicate in the connected pores of the scaffold. Therefore, the scaffold with a large amount of free water can facilitate cellular activity and nutrient circulation.

Moreover, P17Ty scaffolds can maintain good resilience after immersing in PBS at 37°C for 20 days. The stress–strain curves of cyclic compression of the scaffolds are shown in Figure 2E. For the first compression, free water stored in the pores of the scaffold is pressed out so that the stress–strain curve of the first compression does not coincide with the curve of the second compression. However, the compression curves of P17Ty scaffolds for the second and the tenth cycle almost overlap, especially for the cross-linked P17T5, P17T10 and P17T15 scaffolds. After 10 compression cycles, almost all P17Ty scaffolds retain a connected pore structure with little structural deformation and damage (Figure 2F). The above results indicate that the P17Ty scaffolds have excellent structural and mechanical stability, representing promising behavior as tissue engineering scaffolds.

Meanwhile, the above results also well proved the superiority of our strategy. The modulus of WBPU is modulated through the manipulation of phase separation and water permeation via crosslinking. Specifically, amorphous aliphatic polycarbonate and TMP are incorporated into PCL-based WBPU to disrupt interchain hydrogen bonding within polymer segments, suppress microphase separation, impede the crystallization process and bolster covalent crosslinking. Consequently, by altering the crosslinking density and the extent of microphase separation in WBPU, the modulus and water permeability of the material can be effectively modulated.

### Modulus-dependent cell behavior in WBPU scaffolds

**Figure 3.**
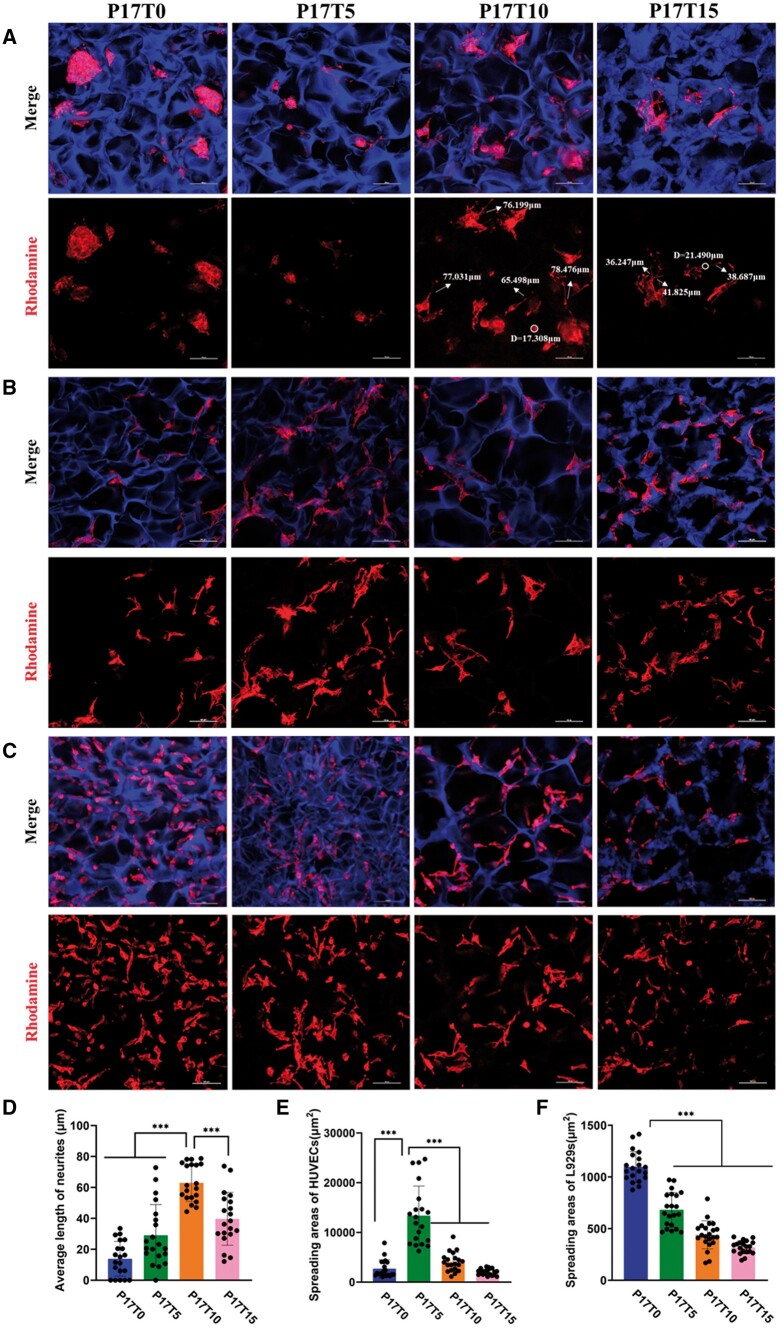
(**A**) Cell morphology of PC12 cells in the P17Ty scaffolds. (**B**) Cell morphology of HUVEC cells in the P17Ty scaffolds. (**C**) Cell morphology of L929 cells in the P17Ty scaffolds. F-actin is stained with rhodamine-phalloidin, red; cell nuclei are stained with DAPI, blue (scale bars were 100 μm) (**D**) Statistics of length of PC12 nerves in scaffolds with different modulus (*n* = 25). (**E**) statistics of HUVEC cells spreading area in scaffolds with different modulus (*n* = 25). (**F**) statistics of L929 cells spreading area in scaffolds with different modulus (*n* = 25). ***P < 0.001.

It is well established that cell behavior strongly depends on the modulus of scaffolds [49]. Central nervous tissue is one of the softest tissues of the human organism, and scaffolds with a low modulus usually facilitate the growth of nervous cells [50]. PC12 cells derived from mouse pheochromocytoma can be used as neural precursor model cells in the undifferentiated state, and their differentiation and growth of neurites can be induced by various stimulations in soft scaffolds. After approving the non-cytotoxicity of the degradation products of the P17Ty scaffolds (Supplementary Figure S4), the growth of PC12 cells in the scaffolds is examined by confocal microscopy, as shown in Figure 3A. Since the PU scaffolds readily absorb a blue dye, the presented scaffolds exhibit a vivid 3D porous morphology with blue color. The PC12 cells can grow into the pores of the P17Ty scaffolds, especially in the soft P17T10 and P17T15 scaffolds. However, the PC12 neurons in the relatively stiff P17T0 scaffold show a spherical agglomeration of cells, hindering their spreading. With a decrease in the material modulus, PC12 cells gradually spread out and differentiate into neurites. By measuring the length of differentiated PC12 nerves, the average length of neurites in the P17T10 scaffold is significantly higher than other groups (Figure 3D), revealing the best ability to induce PC12 differentiation for the relatively soft P17T10 scaffold.

Furthermore, we verified that HUVEC endothelial cells show the most favorable adhesion and proliferation on the P17T5 scaffolds with a moderate modulus (Figure 3B and E) because endothelial cells belong to a group of soft tissue cells with a relative preference toward medium modulus [51]. In contrast, L929 fibroblasts show the most favorable adhesion and proliferation on the P17T0 scaffolds with a relatively high modulus (Figure 3C and F) because fibroblasts belong to a group of soft tissue cells that prefer harder substrate [1]. These results indicate that the gradient modulus of the P17Ty scaffolds can adapt to different cells for various tissue regeneration purposes.

**Figure 4.**
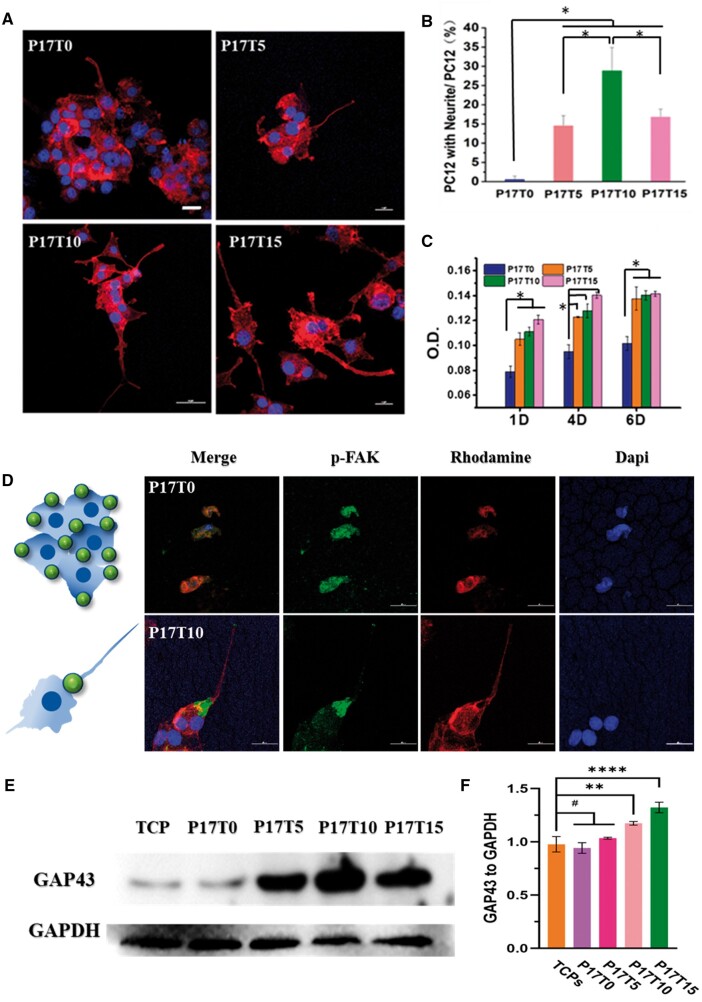
(**A**) Morphology of PC12 on the P17Ty films, scale bars: 20 μm; (**B**) the cell differentiation rate of PC12 on the P17Ty films; (**C**) proliferation of PC12 cells on the P17Ty films after 1, 4 and 6 days. (**D**) Immunofluorescence of PC12 on PU films (p-FAK: green; F-actin: red; nucleus: blue), the green spot in the cartoon picture represents the anchoring spot by adhesion proteins, scale bars: 25 μm; (**E**) WB results of PC12 on the P17Ty films. (**F**) relative expression of GAP-43 on P17Ty films. *P < 0.05, # no significant.

Since cells are difficult to quantify in the 3D porous scaffolds, the P17Ty films with the same components were prepared to co-culture with the representative PC12 cells for quantitative analysis. Similar to the mechanical tendency of the P17Ty scaffolds, the P17Ty films after water absorption also show a decrease in Young’s modulus from 500 kPa for P17T0 to 250 kPa for P17T15 as the crosslinking degree increases (Supplementary Figure S5). Notably, neuronal cells prefer softer materials. As shown in Figure 4A, PC12 cells are poorly dispersed on the stiffest P17T0 film, exhibiting round agglomerated shape. With a decrease in the samples’ stiffness, PC12 cells gradually disperse and extend, finally differentiating into long neurites on P17T10 and P17T15 films. According to the statistical results with Image J, the cell differentiation rate and spreading area on softer samples are significantly higher than for the stiffest P17T0 sample (Figure 4B). Moreover, the proliferation of PC12 cells on the P17Ty films was measured after 1, 4 and 6 days. The PC12 cell number on the softer P17T10 and P17T15 films is always higher than on the stiffest P17T0 (Figure 4C).

P-FAK (Tyr397) is a phosphorylated adhesion kinase that can be used to characterize the cell adhesion site on the material and even the cell migration direction. As shown in Figure 4D, when PC12 cells are on the stiffest P17T0 film, excessive adhesion proteins are activated, hindering cell diffusion and further inhibiting cell differentiation. There have been similar reports before [52, 53]; when cells grow on stiff materials, they tend to produce many adhesive spots. In contrast, the formation of adhesive spots is inhibited on the softer P17T10 film. When PC12 cells grow on a material with a proper modulus, more gelling kinase is activated in the root systems of neurites for fixing long neurites. Then, the neurites extend forward and crawl with a weak anchoring effect until a strong anchoring event occurs between the neurite terminal part and the material to stop the extension.

Growth-associated protein-43 (GAP-43), located on the growth cone and involved in the extension of the axon membrane, is highly expressed during the development of the nervous system and nerve regeneration. As shown in Figure 4E and F, a high expression of GAP-43 protein is present in the P17Ty samples, compared with the reference TCP (tissue culture polystyrene). Moreover, the analysis of the gray part shows that PC12 neurons exhibit the highest expression of the GAP-43 protein on the P17T15 film.

The described cell behaviors demonstrated that the modulus of the P17Ty films can be used to regulate the differentiation of PC12 neurons and even affect the axon growth of PC12 neurons. Furthermore, we also clearly observed that L929 fibroblasts show the best adhesion and proliferation on the P17T0 scaffolds with a relatively high modulus while HUVEC endothelial cells show the best growth state on the P17T5 films with a moderate modulus (Supplementary Figure S6).

### Modulus-dependent immune response of microglial BV2 cells in WBPU scaffolds

Neuroinflammation is primarily mediated by activated microglia, often triggered by the damaged tissue itself or by the rejection response to implants introduced during the repair process, thereby inducing the production of inflammatory enzymes, cytokines and transcription factors. During the process of neural tissue regeneration, microglial cells act as sensors and recruit various cells to the site of damaged nerve tissue for repair. Therefore, it is very significant to explore the influence of mechanically adaptable scaffolds on microglial cells. Microglial BV2 is a cell line that can represent primary microglia. Lipopolysaccharide (LPS) is an antigen on the surface of Gram-negative bacteria. BV2 cells stimulated by LPS can simulate microglia in an inflammatory environment. Microglial cells can adapt to the environment by exhibiting different activation phenotypes with significant morphological changes [54]. As shown in Supplementary Figure S7, BV2 microglial cells in the resting state (M0) maintain a small size with no specific shape (the TCP sample without LPS). When BV2 is activated by LPS and other cytokines, acting as an inflammatory cell model (M1), the cell body grows and the cell edge becomes rough (the TCP sample with LPS in Supplementary Figure S7) [55, 56]. When BV2 is in the anti-inflammatory state (M2), the cells exhibit an elongated cell body with roundish or spindle shape.

**Figure 5.**
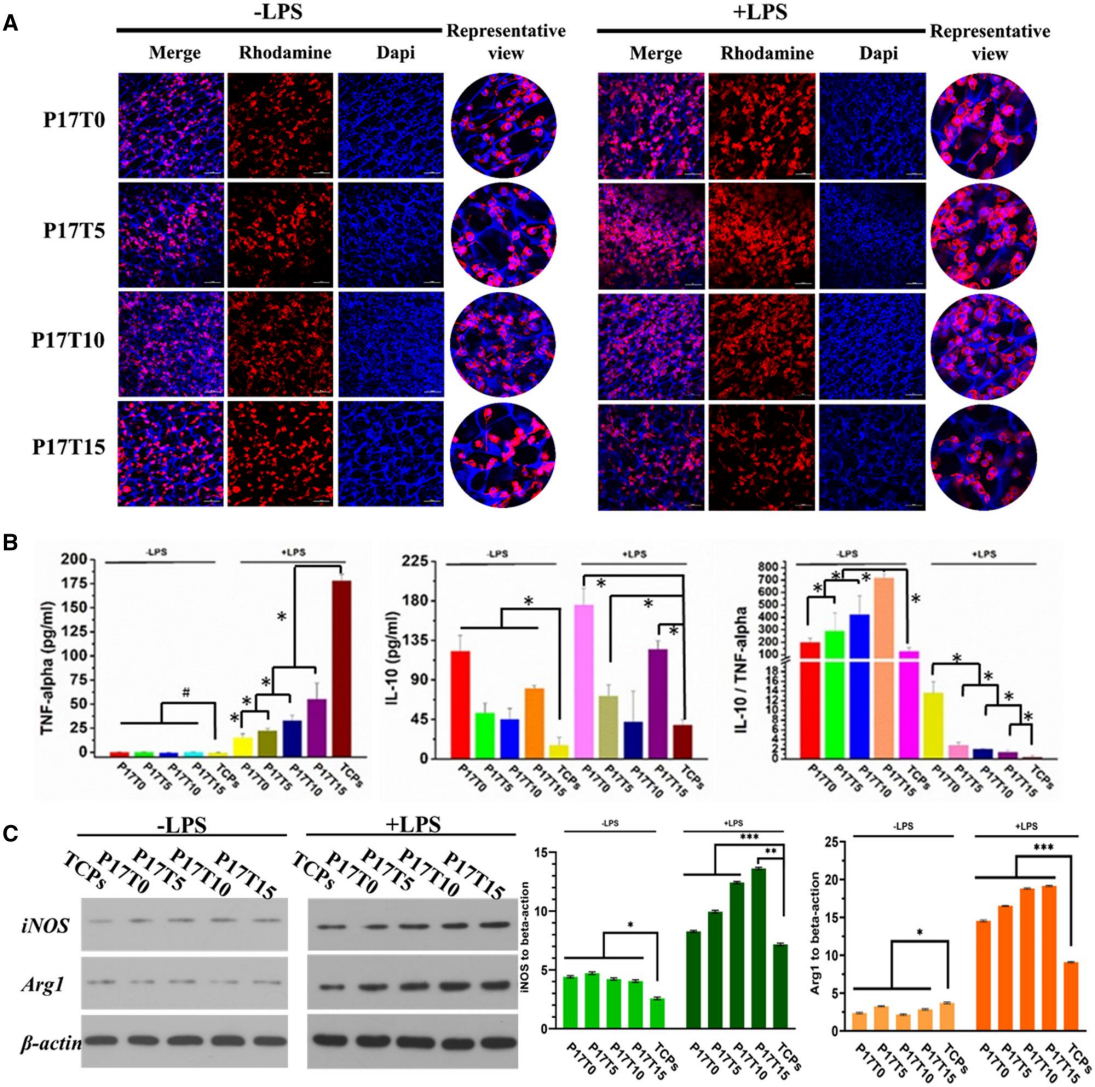
(**A**) Morphology of BV2 on the P17Ty scaffolds with (+) and without (−) LPS, scale bars: 100 μm; (**B**) the concentration of TNF-alpha and IL-10 produced by BV2 on the P17Ty scaffolds, and the ratio of IL-10 to TNF-alpha; (**C**) WB results and the protein expression of BV2 on the P17Ty scaffolds. *P < 0.05, **P < 0.01, ***P < 0.001, # no significant.

Figure 5A shows the morphology of BV2 cells cultured in the P17Ty scaffolds. Without LPS stimulation, the cells on the scaffold are mostly round, indicating the resting state M0. After LPS stimulation, some spindle-shaped cells appear, but most are still round on scaffolds, indicating low inflammation. Most spindle cells are observed on the P17T0 scaffold. The quantitative cytokine secretion of the BV2 cells on scaffolds is plotted in Figure 5B. Consistent with the resting state, BV2 cells excrete very little proinflammatory cytokine TNF-alpha on the P17Ty scaffolds without LPS stimulation. After the addition of LPS, the TNF-alpha excreted by BV2 cells significantly increases due to the inflammatory environment, showing a decreasing trend with the increasing modulus of scaffolds. Moreover, the concentration of TNF-alpha excreted on all P17Ty scaffolds is significantly lower than that of the control samples. In contrast, the BV2 cells on the P17Ty scaffolds excrete more anti-inflammatory cytokines IL-10 than the control samples, regardless of the LPS stimulation. By calculating the ratio of IL-10 to TNF-alpha, the anti-inflammatory effect of scaffolds increases as the modulus decreases when no LPS inflammation is stimulated. After LPS stimulation, the anti-inflammatory effect of scaffolds increases with the modulus. In both cases, the anti-inflammatory effect of all scaffolds is higher than that of the control sample. Moshayedi *et al.* [57] reported that unactivated microglia cells upregulate inflammation-related cytokines in rigid materials compared with soft materials. During *in vivo* material implantation, microglia can be activated by a rigid implant material, rapidly enveloping the implant material, forming chronic inflammation, and possibly recruiting and activating astrocytes to induce glial scar. This is one of the reasons why the use of soft scaffolds for central nerve repair is needed. In contrast, Li *et al.* [20] found that the anti-inflammatory effect of LPS-activated macrophages was more likely to be activated on rigid materials. Since BV2 cells are similar to macrophages in the central nervous system, our results are consistent with the above reports.

Inflammatory protein iNOS and anti-inflammatory protein Arg1 were further used to confirm the protein expression for BV2 cells in different scaffolds, as shown in Figure 5C. Without LPS stimulation, there is low expression of iNOS and Arg1. When the inflammatory environment is applied to BV2 cells, the expression of both iNOS and Arg1 increases, but more significantly for Arg1, indicating that the P17Ty scaffolds have a certain anti-inflammatory ability. Remarkably, softer P17T10 and P17T15 scaffolds exhibit better anti-inflammatory ability than stiffer P17T0 and P17T5 scaffolds.

In combination with the results of co-culturing with PC12 cells and the inflammatory response for the scaffolds, the soft P17T10 scaffold can be selected to better repair the central nervous tissue with natural low modulus.

### *In vivo* brain tissue regeneration of the TBI model by WBPU scaffolds

**Figure 6.**
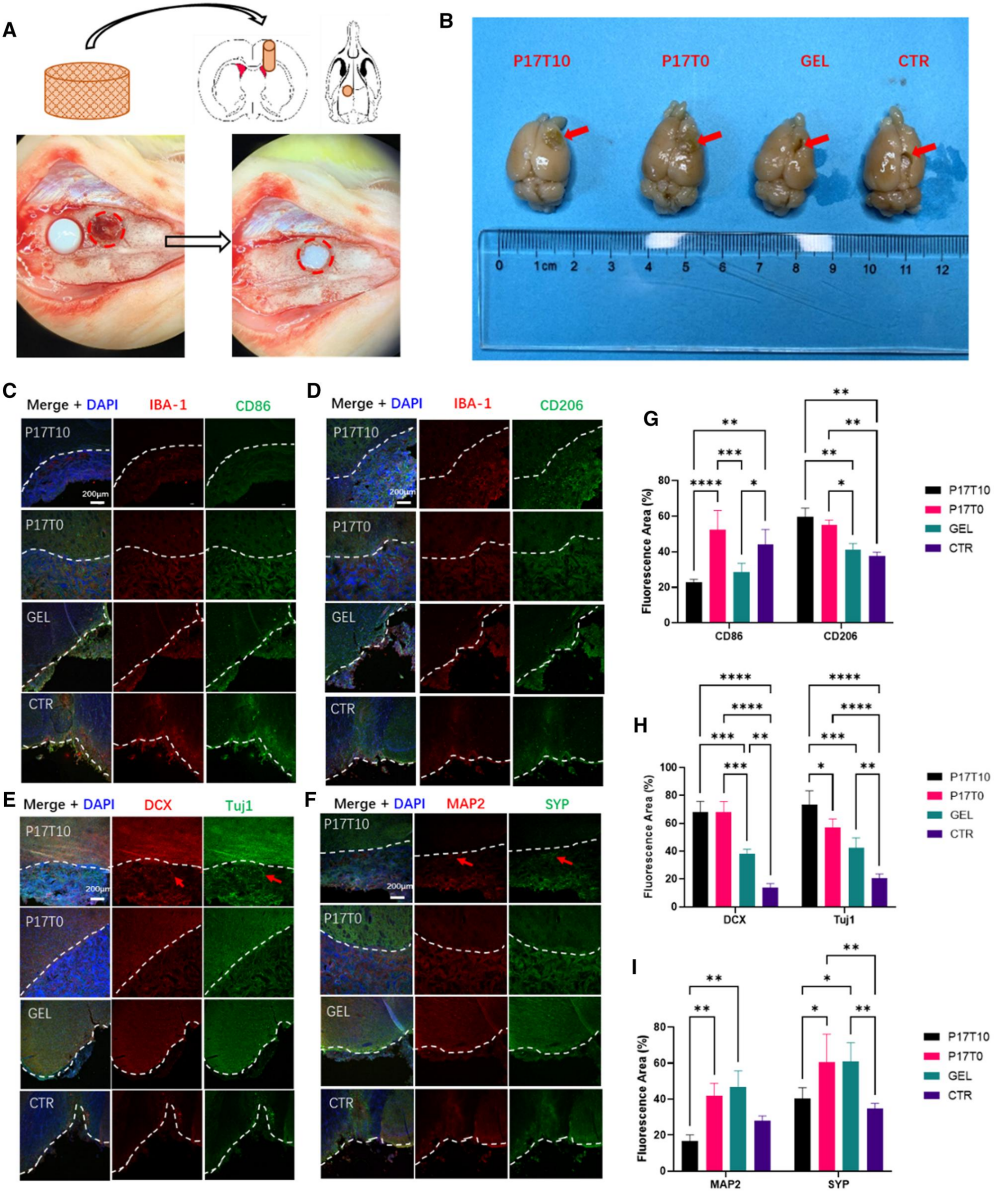
(**A**) Schematic procedure of the TBI model showing a scaffold (brownish cylinder) implanted into the traumatic brain cavity and typical digital photographs of brain defects and implanting surgery. (**B**) Representative photographs of damaged brain tissue treated with different scaffolds post-injury on the 14th day. The damaged zones are pointed out by arrows. GEL and CTR represent the PU hydrogel and control groups, respectively. (**C**) Microglia (IBA-1, red) and CD 86 cells (green) on scaffolds and control groups. Notably, all the implanting materials are below the white line. (**D**) Microglia (IBA-1, red) and CD 206 cells (green) on scaffolds and control groups. Microglia and immune cells are mainly located in the inner pores of the scaffolds but located on the boundary of traumatic cavity lesions in the hydrogel and control groups. (**E**) Endogenous neural progenitor cells (NPCs) (DCX, red) and new neurons (Tuj1, green) in the scaffolds and control groups. Especially some new neurons (marked by the arrow) are located inside the P17T10 scaffold (below the white line). (**F**) Survival of mature neurons (MAP2, red) and neuronal neurites containing synaptophysin (SYP, green) in the scaffolds and control groups. Survival of mature neurons and neurites (marked by arrow) appearing in traumatic cavity lesions and scaffold pores; scale bars: 200 μm. (**G**) Fluorescence area of CD 86 cells and CD 206 cells on scaffolds and control groups. (**H**) Fluorescence area of DCX and Tuj1 on scaffolds and control groups. (**I**) Fluorescence area of MAP2 and SYP on scaffolds and control groups. *P < 0.05, **P < 0.01, ***P < 0.001 and ****P < 0.0001.

As a representative and challenging application in brain tissue regeneration after the TBI model, P17T0 and P17T10 scaffolds with relatively high and low moduli, respectively, were selected for repair research. Meanwhile, a traditional biodegradable PU hydrogel with a modulus of 7.5 ± 1.2 kPa and a control group (treated with sterilized PBS) were also studied. As shown in Figure 6A, the TBI model was established by operating a circular bone window with a diameter of 8 mm and removing a 5 mm × 5 mm × 4 mm brain tissue in the rat motor center zone through microsurgery, which accounted for 4.5% of the rat’s whole brain volume. Then, the tailored scaffolds were implanted into the lesion cavity. Animals were sacrificed 14-day post-injury to evaluate the repair progress through immunohistochemistry. Representative photographs of the injured brain tissue treated with different scaffolds post-injury on the 14th day are shown in Figure 6B. Scaffold-treated rats exhibit a notable reduction of the traumatic brain cavity volume with no tissue damage. Visibly decreased hemosiderin deposition appears in the scaffold-implanted groups; in contrast, an obvious traumatic cavity with missing brain tissue appears in the control group, and a collapsed cavity with severe hydrogel degradation is observed in the PU hydrogel group. In general, the engineered scaffolds provide a stable framework for brain tissue regeneration, but the hydrogel has a too rapid degradation rate to effectively support the regeneration.

Upon TBI, immune cells are immediately activated to regulate neuronal regeneration and tissue remodeling [58–61]. Therefore, the distribution and functional status of microglia/macrophages were first studied. As shown in Figure 6C and D, 14-day post-injury, microglia/macrophages mainly appear in the inner pores of the scaffolds, while they are gathered on the edges of the traumatic cavity lesions for the PU hydrogel and control groups. The expression of inflammatory CD 86 cells (M1-like cells) is more obvious in the control group, and a decrease in CD 86 cells is detected in the P17T10 scaffold and PU hydrogel. In addition, a lower inflammatory expression in the softer P17T10 scaffold is observed compared with the P17T0 scaffold group (Figure 6G). In contrast, the density of anti-inflammatory CD206 cells (M2-like cells) mainly distributed in pores of P17T10 is the highest. Compared with the scaffolds, the hydrogel group shows a stronger inflammatory response but better than the control group. These results indicate that the low-modulus P17T10 scaffold suppresses the inflammatory response of traumatic cavity lesions accompanied by dispersing of the immune cells in 3D pores to promote the differentiation of M2-like cells. This is consistent with the results of *in vitro* experiments.

During neural regeneration, the activation and immigration of endogenous NPCs are vital. As shown in Figure 6E, a large number of activated NPCs migrate to the pores inside the scaffolds P17T0 and P17T10, especially at the junction between the scaffold and the damaged area, and differentiate into new neurons. The numbers of NPCs and new neurons in the softer P17T10 scaffold are larger than in the P17T0 scaffold. For the PU hydrogel treatment group, a sharp boundary of the traumatic cavity lesions appears, and few hydrogels can be found because of the severe degradation and the mismatched pore size of the hydrogel for cell infiltration. Thus, the NPCs are activated in the injury zone and only a few new neurons are located on the boundary of lesions. In the control group, a few NPCs are activated and new neurons are very scarce on the boundary of lesions. Therefore, the expression of both DCX and Tuj1 for P17T10 and P17T0 scaffolds are significantly higher than those for the PU hydrogel and control group (Figure 6H). After the migration of active NPCs, the regeneration of mature neurons (MAP2) and neuronal neurites containing synaptophysin (SYP) is required to restore brain function. As shown in Figure 6F, the mature neurons and neuronal neurites notably infiltrate the pores of the scaffolds. Especially new neurons with neurites appear in the surrounding new tissue and stretch across the interface into the interior part of P17T10 scaffolds. Although a relatively high expression of MAP2 and SYP also exists in the hydrogel group (Figure 6I), these cells are dispersed in the traumatic cavity lesions and cannot achieve structural and functional regeneration of the brain tissue. In the control group, the mature neurons and neuronal neurites are mainly gathered in the surrounding traumatic tissue.

According to the above results, the WBPU scaffolds serve as a regenerative niche to guide the migration and differentiation of endogenous NPCs into mature neurons and neuronal neurites from outside the scaffolds to the internal pores. Meanwhile, the immune cells are mildly activated and recruited to immigrate into the pores of the scaffolds so that a low inflammatory response occurs in the scaffold-implanted groups. In particular, the low-modulus P17T10 scaffold is optimal for brain tissue repair, demonstrating the lowest inflammatory stimulation and the highest neuronal regeneration to restore the brain tissue. In contrast, the PU hydrogel fails to restore the traumatic brain tissue because of severe degradation and mismatched pore size for cell infiltration so that many microglia cells concentrate on the boundary of traumatic cavity lesions.

In addition, after 8 weeks of TBI, motor function analysis was performed on rats using the Bederson scale. The results revealed that rats in the scaffold implantation groups had obvious functional recovery compared with those in the control group (Supplementary Figure S8). The P17T10 scaffold group presented the highest cure rate that almost all the rats recovered the motor function. Therefore, the proposed P17T10 scaffold with a low modulus is a promising biomaterial to restore the traumatic brain cavity, both structurally and functionally.

## Conclusions

Amorphous PCDL and the TMP cross-linker were introduced to PCL-based WBPU scaffolds in a facile and effective strategy to adjust the modulus via interrupting crystallization and increasing the degree of covalent cross-linking. The cross-linking manipulation of phase separation and water infiltration induced that the modulus of the WBPUs exhibited an abnormal gradual decrease with the cross-linking degree. A series of WBPU scaffolds with gradient moduli was obtained to adapt various tissue cells and regulate the inflammatory response. As a representative application of brain tissue regeneration after the TBI model, we demonstrated that the softer P17T10 scaffold has effective brain tissue repair effect, including central nerve regeneration, which was obviously better than the traditional biodegradable PU hydrogel or the stiffer P17T0 scaffold. Therefore, the development of a mechanically adaptable scaffold may be favorable for activating the migration of endogenous progenitor cells, stimulating a positive immune response and unlocking the body’s innate regeneration power. These findings offer a promising strategy for designing mechanically adjustable scaffolds for versatile applications in soft tissue regeneration.

## Supplementary Material

rbae111_Supplementary_Data

## References

[1] Ma Y , Lin M, Huang G, Li Y, Wang S, Bai G, Lu T, Xu F. 3D spatiotemporal mechanical microenvironment: a hydrogel-based platform for guiding stem cell fate. Adv Mater 2018;30:1705911. 10.1002/adma.201705911 30063260

[2] Wang S , Zhu C, Zhang B, Hu J, Xu J, Xue C, Bao S, Gu X, Ding F, Yang Y, Gu X, Gu Y. BMSC-derived extracellular matrix better optimizes the microenvironment to support nerve regeneration. Biomaterials 2022;280:121251. 34810037 10.1016/j.biomaterials.2021.121251

[3] Li Y , Xiao Y, Liu C. The horizon of materiobiology: a perspective on material-guided cell behaviors and tissue engineering. Chem Rev 2017;117:4376–421. 28221776 10.1021/acs.chemrev.6b00654

[4] Ye K , Wang X, Cao L, Li S, Li Z, Yu L, Ding J. Matrix stiffness and nanoscale spatial organization of cell-adhesive ligands direct stem cell fate. Nano Lett 2015;15:4720–9. 26027605 10.1021/acs.nanolett.5b01619

[5] Wang Y , Liang R, Lin J, Chen J, Zhang Q, Li J, Wang M, Hui X, Tan H, Fu Q. Biodegradable polyurethane nerve guide conduits with different moduli influence axon regeneration in transected peripheral nerve injury. J Mater Chem B 2021;9:7979–90. 34612287 10.1039/d1tb01236c

[6] Li Y , Zhou M, Zheng W, Yang J, Jiang N. Scaffold-based tissue engineering strategies for soft–hard interface regeneration. Regen Biomater 2023;10:rbac091. 36683751 10.1093/rb/rbac091 PMC9847541

[7] Jin W , Wu H, Shi J, Hu Z, Zhou Y, Chen Z, Shao C, Tang R, Xie Z. Biomimetic mineralized collagen scaffolds enhancing odontogenic differentiation of hDPSCs and dentin regeneration through modulating mechanical microenvironment. Chem Eng J 2023;460:141800.

[8] Dey K , Roca E, Ramorino G, Sartore L. Progress in the mechanical modulation of cell functions in tissue engineering. Biomater Sci 2020;8:7033–81. 33150878 10.1039/d0bm01255f

[9] Dong Z , Han W, Jiang P, Hao L, Fu X. Regulation of mitochondrial network architecture and function in mesenchymal stem cells by micropatterned surfaces. Regen Biomater 2024;11:rbae052. 38854681 10.1093/rb/rbae052 PMC11162196

[10] Vining KH , Mooney DJ. Mechanical forces direct stem cell behaviour in development and regeneration. Nat Rev Mol Cell Biol 2017;18:728–42. 29115301 10.1038/nrm.2017.108 PMC5803560

[11] Babu S , Chen I, Vedaraman S, Gerardo-Nava J, Licht C, Kittel Y, Haraszti T, Russo J, Laporte L. How do the local physical, biochemical, and mechanical properties of an injectable synthetic anisotropic hydrogel affect oriented nerve growth? Adv Funct Mater 2022;32:2202468.

[12] Unal DB , Caliari SR, Lampe KJ. 3D hyaluronic acid hydrogels for modeling oligodendrocyte progenitor cell behavior as a function of matrix stiffness. Biomacromolecules 2020;21:4962–71. 33112592 10.1021/acs.biomac.0c01164

[13] Meyer N , Bax D, Beck J, Best CR. S. Adjusting the physico-chemical properties of collagen scaffolds to accommodate primary osteoblasts and endothelial cells. Regen Biomater 2023;10:rbad015. 36937897 10.1093/rb/rbad015 PMC10019812

[14] Hu T , Shi M, Zhao X, Liang Y, Bi L, Zhang Z, Liu S, Chen B, Duan X, Guo B. Biomimetic 3D aligned conductive tubular cryogel scaffolds with mechanical anisotropy for 3D cell alignment, differentiation and in vivo skeletal muscle regeneration. Chem Eng J 2022;428:131017.

[15] Gefen A , Margulies SS. Are in vivo and in situ brain tissues mechanically similar? J Biomech 2004;37:1339–52. 15275841 10.1016/j.jbiomech.2003.12.032

[16] Leipzig ND , Shoichet MS. The effect of substrate stiffness on adult neural stem cell behavior. Biomaterials 2009;30:6867–78. 19775749 10.1016/j.biomaterials.2009.09.002

[17] Ali S , Wall IB, Mason C, Pelling AE, Veraitch FS. The effect of Young’s modulus on the neuronal differentiation of mouse embryonic stem cells. Acta Biomater 2015;25:253–67. 26159105 10.1016/j.actbio.2015.07.008

[18] Stukel JM , Willits RK. The interplay of peptide affinity and scaffold stiffness on neuronal differentiation of neural stem cells. Biomed Mater 2018;13:024102. 29133625 10.1088/1748-605X/aa9a4b

[19] Chesnokova V , Pechnick RN, Wawrowsky K. Chronic peripheral inflammation, hippocampal neurogenesis, and behavior. Brain Behav Immun 2016;58:1–8. 26802985 10.1016/j.bbi.2016.01.017 PMC4956598

[20] Taraballi F , Sushnitha M, Tsao C, Bauza G, Liverani C, Shi A, Tasciotti E. Biomimetic tissue engineering: tuning the immune and inflammatory response to implantable biomaterials. Adv Healthc Mater 2018;7:1800490. 10.1002/adhm.201800490 29995315

[21] Chuang Y-C , Chang H-M, Li C-Y, Cui Y, Lee C-L, Chen C-S. Reactive oxygen species and inflammatory responses of macrophages to substrates with physiological stiffness. ACS Appl Mater Interfaces 2020;12:48432–41. 33064443 10.1021/acsami.0c16638

[22] Li Z , Bratlie KM. How cross-linking mechanisms of methacrylated gellan gum hydrogels alter macrophage phenotype. ACS Appl Bio Mater 2019;2:217–25. 10.1021/acsabm.8b00562 35016344

[23] Zhuang Z , Zhang Y, Sun S, Li Q, Chen K, An C, Jeroen WL, van den Beucken JJP, Wang H. Control of matrix stiffness using methacrylate–gelatin hydrogels for a macrophage-mediated inflammatory response. ACS Biomater Sci Eng 2020;6:3091–102. 33463297 10.1021/acsbiomaterials.0c00295

[24] Simon DW , McGeachy MJ, Bayır H, Clark RSB, Loane DJ, Kochanek PM. The far-reaching scope of neuroinflammation after traumatic brain injury. Nat Rev Neurol 2017;13:171–91. 28186177 10.1038/nrneurol.2017.13 PMC5675525

[25] Zhong J , Yang Y, Liao L, Zhang C. Matrix stiffness-regulated cellular functions under different dimensionalities. Biomater Sci 2020;8:2734–55. 32322859 10.1039/c9bm01809c

[26] Nguyen DHT , Utama RH, Tjandra KC, Suwannakot P, Du EY, Kavallaris M, Tilley RD, Gooding JJ. Tuning the mechanical properties of multiarm RAFT-based block copolyelectrolyte hydrogels via ionic cross-linking for 3D cell cultures. Biomacromolecules 2023;24:57–68. 36514252 10.1021/acs.biomac.2c00632

[27] Yeh Y-C , Ouyang L, Highley CB, Burdick JA. Norbornene-modified poly(glycerol sebacate) as a photocurable and biodegradable elastomer. Polym Chem 2017;8:5091–9.

[28] Bai M , Xie J, Liu X, Chen X, Liu W, Wu F, Chen D, Sun Y, Li X, Wang C, Ye L. Microenvironmental stiffness regulates dental papilla cell differentiation: implications for the importance of fibronectin–paxillin–β–catenin axis. ACS Appl Mater Interfaces 2018;10:26917–27. 30004214 10.1021/acsami.8b08450

[29] Pek YS , Wan AC, Ying JY. The effect of matrix stiffness on mesenchymal stem cell differentiation in a 3D thixotropic gel. Biomaterials 2010;31:385–91. 19811817 10.1016/j.biomaterials.2009.09.057

[30] Ding X , Zhao H, Li Y, Lee A-L, Li Z, Fu M, Li C, Yang Y-Y, Yuan P. Synthetic peptide hydrogels as 3D scaffolds for tissue engineering. Adv Drug Deliv Rev 2020;160:78–104. 33091503 10.1016/j.addr.2020.10.005

[31] Tseng TC , Tao L, Hsieh FY, Wei Y, Chiu I-M, Hsu S-h An injectable, self-healing hydrogel to repair the central nervous system. Adv Mater 2015;27:3518–24. 25953204 10.1002/adma.201500762

[32] Ma Y , Han T, Yang Q, Wang J, Feng B, Jia Y, Wei Z, Xu F. Viscoelastic cell microenvironment: hydrogel-based strategy for recapitulating dynamic ECM mechanics. Adv Funct Mater 2021;31:2100848.

[33] Lü X , Zhang H, Huang Y, Zhang Y. A proteomics study to explore the role of adsorbed serum proteins for PC12 cell adhesion and growth on chitosan and collagen/chitosan surfaces. Regen Biomater 2018;5:261–73. 30338124 10.1093/rb/rby017 PMC6184651

[34] Liang Y , Qiao L, Qiao B, Guo B. Conductive hydrogels for tissue repair. Chem Sci 2023;14:3091–116. 36970088 10.1039/d3sc00145h PMC10034154

[35] Tran RT , Choy WM, Cao H, Qattan I, Chiao J-C, Ip WY, Yeung KWK, Yang J. Fabrication and characterization of biomimetic multichanneled crosslinked-urethane-doped polyester tissue engineered nerve guides. J Biomed Mater Res A 2014;102:2793–804. 24115502 10.1002/jbm.a.34952 PMC3965663

[36] Hayman MW , Smith KH, Cameron NR, Przyborski SA. Growth of human stem cell-derived neurons on solid three-dimensional polymers. J Biochem Biophys Methods 2005;62:231–40. 15733583 10.1016/j.jbbm.2004.12.001

[37] Chwalek K , Tang-Schomer MD, Omenetto FG, Kaplan DL. In vitro bioengineered model of cortical brain tissue. Nat Protoc 2015;10:1362–73. 26270395 10.1038/nprot.2015.091 PMC4867028

[38] Jiang X , Yu F, Wang Z, Li J, Tan H, Ding M, Fu Q. Fabrication and characterization of waterborne biodegradable polyurethanes 3-dimensional porous scaffolds for vascular tissue engineering. J Biomater Sci Polym Ed 2010;21:1637–52. 20537246 10.1163/092050609X12525750021270

[39] Guo W , Zhao B, Shafiq M, Yu X, Shen Y, Cui J, Chen Y, Cai P, Yuan Z, EL-Newehy M, EL-Hamshary H, Morsi Y, Sun B, Pan J, Mo X. On the development of modular polyurethane-based bioelastomers for rapid hemostasis and wound healing. Regen Biomater 2023;10:rbad019. 36969314 10.1093/rb/rbad019 PMC10038391

[40] Du B , Yin H, Chen Y, Lin W, Wang Y, Zhao D, Wang G, He X, Li J, Li Z, Luo F, Tan H, Fu Q. A waterborne polyurethane 3D scaffold containing PLGA with a controllable degradation rate and an anti-inflammatory effect for potential applications in neural tissue repair. J Mater Chem B 2020;8:4434–46. 32367107 10.1039/d0tb00656d

[41] Chen Y , Long X, Lin W, Du B, Yin H, Lan W, Zhao D, Li Z, Li J, Luo F, Tan H. Bioactive 3D porous cobalt-doped alginate/waterborne polyurethane scaffolds with a coral reef-like rough surface for nerve tissue engineering application. J Mater Chem B 2021;9:322–35. 33242318 10.1039/d0tb02347g

[42] Song N-j , Jiang X, Li J-h, Pang Y, Li J-s, Tan H, Fu Q. The degradation and biocompatibility of waterborne biodegradable polyurethanes for tissue engineering. Chin J Polym Sci 2013;31:1451–62.

[43] Wang Y-C , Fang F, Wu Y-K, Ai X-L, Lan T, Liang R-C, Zhang Y, Trishul NM, He M, You C, Yu C, Tan H. Waterborne biodegradable polyurethane 3-dimensional porous scaffold for rat cerebral tissue regeneration. RSC Adv 2016;6:3840–9.

[44] Zhang F , Wang R, He Y, Lin W, Li Y, Shao Y, Li J, Ding M, Luo F, Tan H, Fu Q. A biomimetic hierarchical structure with a hydrophilic surface and a hydrophobic subsurface constructed from waterborne polyurethanes containing a self-assembling peptide extender. J Mater Chem B 2018;6:4326–37. 32254508 10.1039/c8tb01279b

[45] Hao H , Shao J, Deng Y, He S, Luo F, Wu Y, Li J, Tan H, Li J, Fu Q. Synthesis and characterization of biodegradable lysine-based waterborne polyurethane for soft tissue engineering applications. Biomater Sci 2016;4:1682–90. 27709130 10.1039/c6bm00588h

[46] Bederson JB , Pitts LH, Tsuj M, Nishimura MC, Davis RL, Bartkowski H. Rat middle cerebral artery occlusion: evaluation of the model and development of a neurologic examination. Stroke 1986;17:472–6. 3715945 10.1161/01.str.17.3.472

[47] Hatakeyama T , Tanaka M, Hatakeyama H. Studies on bound water restrained by poly(2-methacryloyloxyethyl phosphorylcholine): comparison with polysaccharide–water systems. Acta Biomater 2010;6:2077–82. 20005309 10.1016/j.actbio.2009.12.018

[48] Iijima M , Hatakeyama T, Hatakeyama H. DSC and TMA studies on freezing and thawing gelation of galactomannan polysaccharide. Thermochim Acta 2012;532:83–7.

[49] Brusatin G , Panciera T, Gandin A, Citron A, Piccolo S. Biomaterials and engineered microenvironments to control Yap/TAZ-dependent cell behaviour. Nat Mater 2018;17:1063–75. 30374202 10.1038/s41563-018-0180-8 PMC6992423

[50] Yi B , Xu Q, Liu W. An overview of substrate stiffness guided cellular response and its applications in tissue regeneration. Bioact Mater 2022;15:82–102. 35386347 10.1016/j.bioactmat.2021.12.005 PMC8940767

[51] Nguyen EH , Zanotelli MR, Schwartz MP, Murphy WL. Differential effects of cell adhesion, modulus and VEGFR-2 inhibition on capillary network formation in synthetic hydrogel arrays. Biomaterials 2014;35:2149–61. 24332391 10.1016/j.biomaterials.2013.11.054 PMC3970236

[52] Zhan X. Effect of matrix stiffness and adhesion ligand density on chondrogenic differentiation of mesenchymal stem cells. J Biomed Mater Res A 2020;108:675–83. 31747107 10.1002/jbm.a.36847

[53] George E , Barai A, Shirke P, Majumder A, Sen S. Engineering interfacial migration by collective tuning of adhesion anisotropy and stiffness. Acta Biomater 2018;72:82–93. 29574184 10.1016/j.actbio.2018.03.016

[54] Toorres-Platas SG , Comeau S, Rachalski A, Bo GD, Cruceanu C, Turecki G, Giros B, Mechawar N. Morphometric characterization of microglial phenotypes in human neocortex. J Neuroinflamm 2014;11:12. 10.1186/1742-2094-11-12 PMC3906907 24447857

[55] Heinrich F , Lehmbecker A, Raddatz BB, Kegler K, Tipold A, Stein VM, Kalkuhl A, Deschl U, Baumgärtner W, Ulrich R, Spitzbarth I. Morphologic, phenotypic, and transcriptomic characterization of classically and alternatively activated canine blood-derived macrophages in vitro. PLoS One 2017;12:e0183572. 28817687 10.1371/journal.pone.0183572 PMC5560737

[56] Jian C , Jiahe W, Jiafu M, Feng S, Gao J. The design criteria and therapeutic strategy of functional scaffolds for spinal cord injury repair. [J]. Biomater Sci 2021;9:4591–606. 34018520 10.1039/d1bm00361e

[57] Moshayedi P , Ng G, Kwok JC, Yeo GS, Bryant CE, Fawcett JM, Franze K, Guck J. The relationship between glial cell mechanosensitivity and foreign body reactions in the central nervous system. Biomaterials 2014;35:3919–25. 24529901 10.1016/j.biomaterials.2014.01.038

[58] Bellver-Landete V , Bretheau F, Mailhot B, Vallières N, Lessard M, Janelle M-E, Vernoux N, Tremblay M-È, Fuehrmann T, Shoichet MS, Lacroix S. Microglia are an essential component of the neuroprotective scar that forms after spinal cord injury. Nat Commun 2019;10:518. 30705270 10.1038/s41467-019-08446-0 PMC6355913

[59] Liu X , Zhang G, Wei P, Zhong L, Chen Y, Zhang J, Chen X, Zhou L. Three-dimensional-printed collagen/chitosan/secretome derived from HUCMSCs scaffolds for efficient neural network reconstruction in canines with traumatic brain injury. Regen Biomater 2022;9:rbac043. 35855109 10.1093/rb/rbac043 PMC9290528

[60] Liu X , Zhang J, Cheng X, Liu P, Feng Q, Wang S, Li Y, Gu H, Zhong H, Chen M, Liangxue Z. Integrated printed BDNF-stimulated HUCMSCs-derived exosomes/collagen/chitosan biological scaffolds with 3D printing technology promoted the remodelling of neural networks after traumatic brain injury. Regen Biomater 2023;10:rbac085. 36683754 10.1093/rb/rbac085 PMC9847532

[61] Latchoumane C-FV , Betancur MI, Simchick GA, Sun MK, Forghani R, Lenear CE, Ahmed A, Mohankumar R, Balaji N, Mason HD, Archer-Hartmann SA, Azadi P, Holmes PV, Zhao Q, Bellamkonda RV, Karumbaiah L. Engineered glycomaterial implants orchestrate large-scale functional repair of brain tissue chronically after severe traumatic brain injury. Sci Adv 2021;7:eabe0207. 33674306 10.1126/sciadv.abe0207 PMC7935369

