# Supplementary material for: Cross-linking manipulation of waterborne biodegradable polyurethane for constructing mechanically adaptable tissue engineering scaffolds
Source: Regen Biomater. 2024 Sep 2;11:rbae111. doi: 10.1093/rb/rbae111 (PMC11422185; doi:10.1093/rb/rbae111)
Supplement: rbae111_Supplementary_Data [file rbae111_supplementary_data.docx]

Supporting information

**Cross-linking manipulation of waterborne** **biodegradable polyurethane for constructing mechanically adaptable tissue engineering scaffolds**

*Nan Sheng^1^, Weiwei Lin^1^, Jingjing Lin^1^, Yuan Feng^1^, Yanchao Wang^2^, Xueling He^3^, Yuanyuan He^1^, Ruichao Liang^2^, Zhen Li1, Jiehua Li1, Feng Luo^1^*, Hong Tan^1^*.*

1. College of Polymer Science and Engineering, State Key Laboratory of Polymer Materials Engineering, Med-X Center of Materials, Sichuan University, Chengdu 610065, China
2. Department of Neurosurgery, West China Hospital, Sichuan University, Chengdu, Sichuan, 610000, China.
3. Laboratory Animal Center of Sichuan University, Chengdu 610041, China

*Address correspondence to E-mail:

[fengluo@scu.edu.cn](mailto:fengluo@scu.edu.cn) (F.L.) and [hongtan@scu.edu.cn](mailto:hongtan@scu.edu.cn) (T.H.)


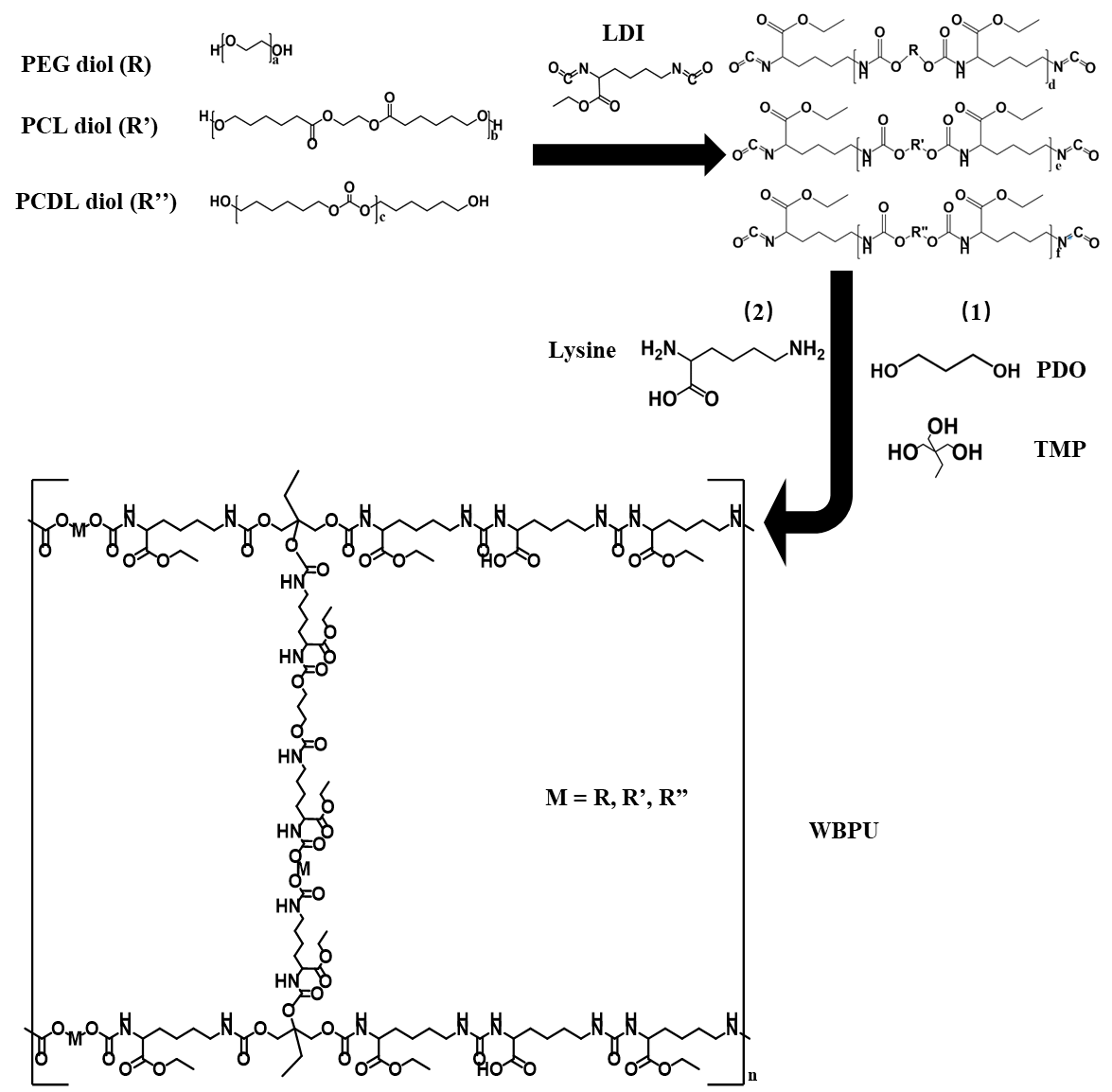


Figure S1. Synthesis process of waterborne biodegradable polyurethane (WBPU).


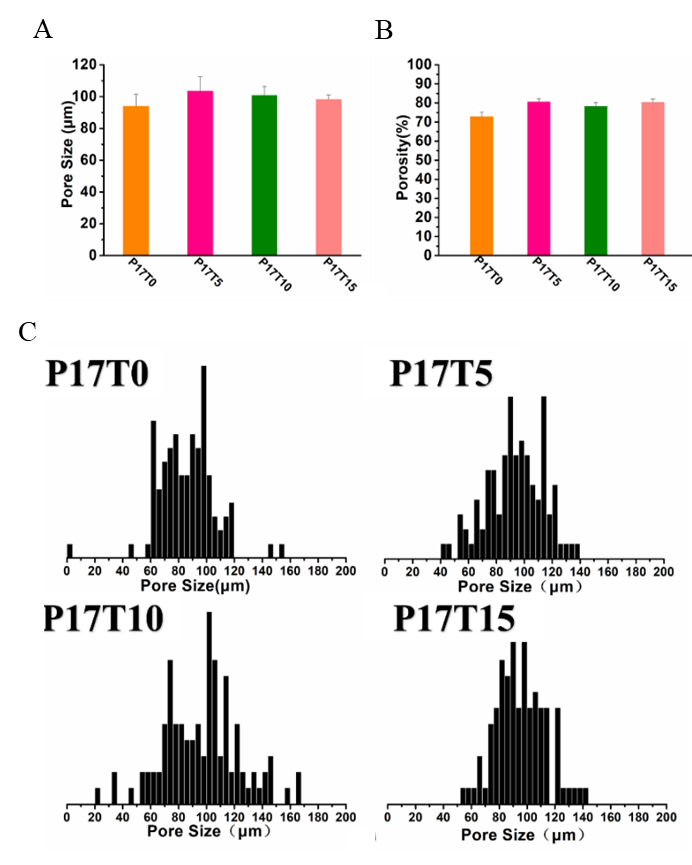


Figure S2. A) Pore size of PxTy scaffolds, B) porosity of PxTy scaffolds, C) distribution of pore size of PxTy scaffolds.


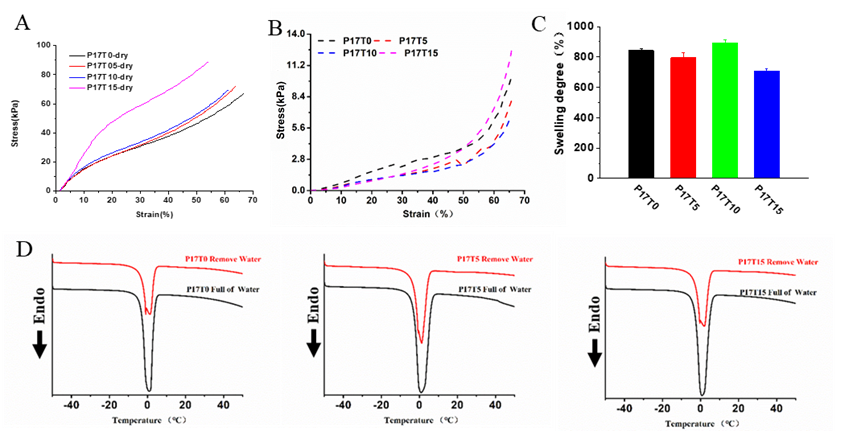


Figure S3. A) The compressive curves of PxTy scaffolds in dry sate; B) The compressive curves of PxTy scaffolds in wet sate. C) Swelling degree of PxTy scaffolds, swelling degree equals the weight of absorbed water in scaffold to that of dry scaffold; D) heating curves of PxTy water-saturated scaffolds and water-unsaturated scaffolds.


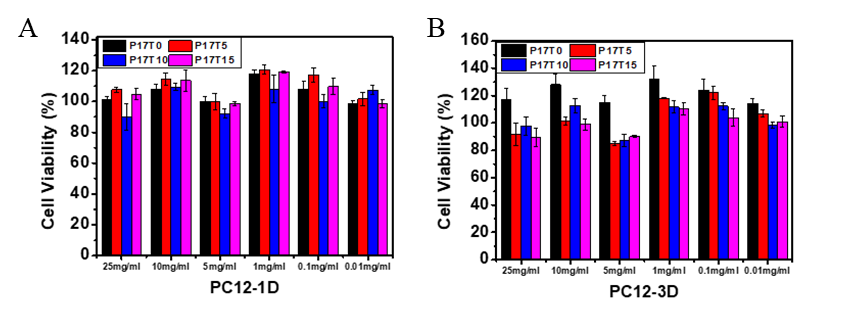


Figure S4. A) The effect of degradation products from PxTy on PC12 viability on 1 day. B) The effect of degradation products from PxTy on PC12 viability on 3 days.


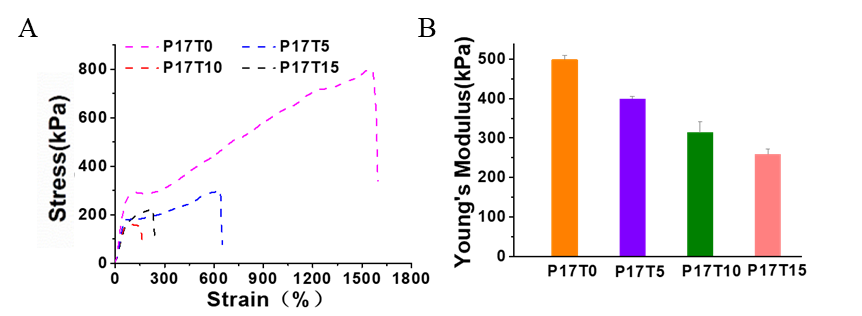


Figure S5. A) Tensile stress-strain curves of P17Ty films after water absorption. B) The Young’s modulus of PxTy films after water absorption.


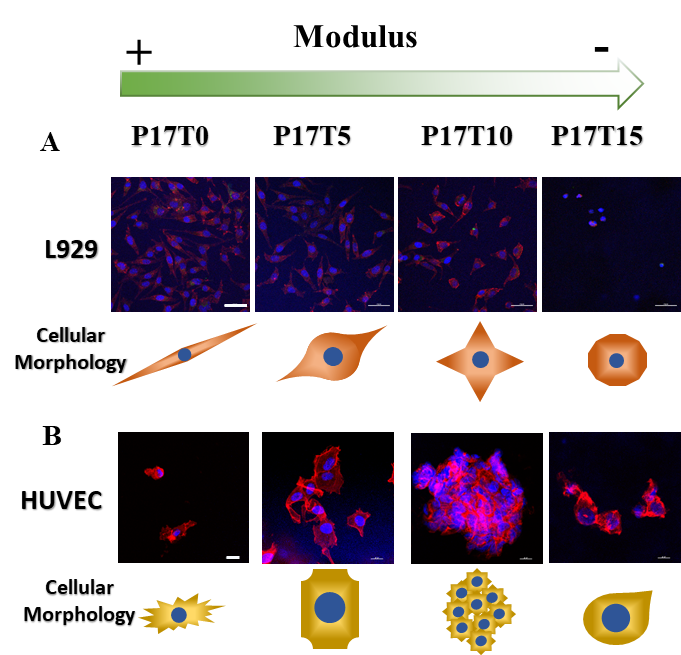


Figure S6. A) Morphology of L929 cells on the P17Ty films, scale bars: 50 μm; B) Morphology of HUVEC on the P17Ty films, scale bars: 20 μm.


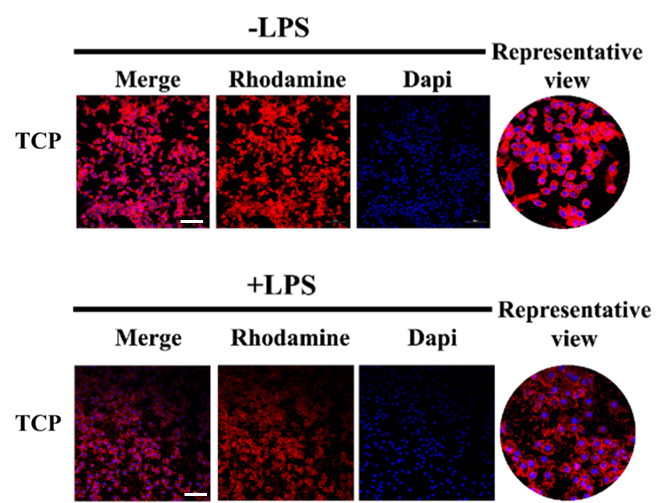


Figure S7. The morphology of BV2 cells on TCPs with (+) and without (-) LPS, scale bars: 100 μm.


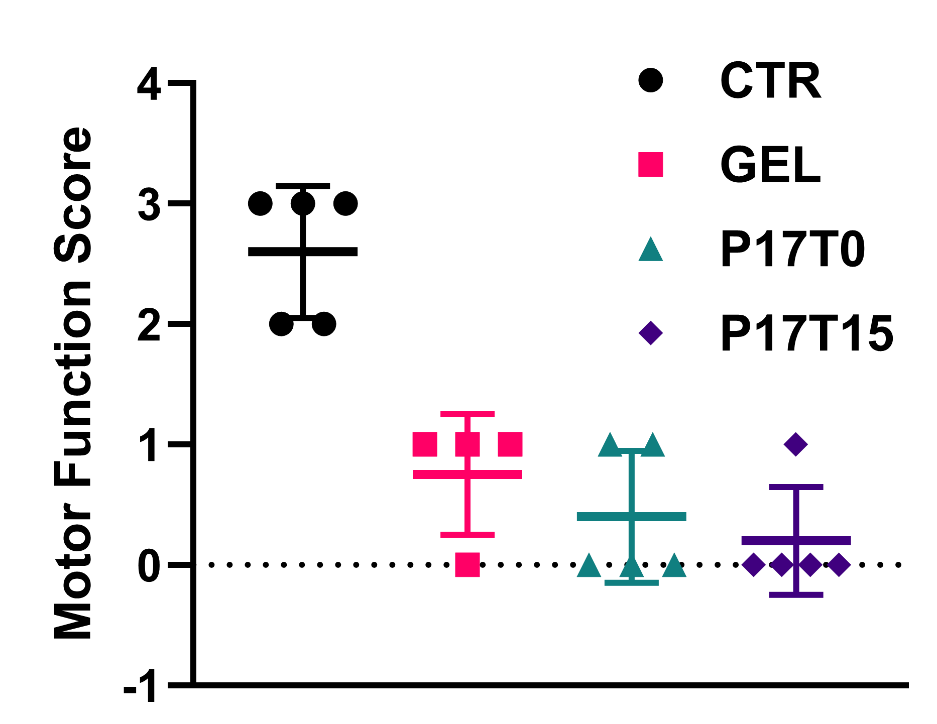


Figure S8. Motor function scores using the Bederson scale for the rats with different treatments after post-injury 8 weeks. Rats with scaffold implantation showed improved functional recovery.
